# A 3D bioprinter platform for mechanistic analysis of tumoroids and chimeric mammary organoids

**DOI:** 10.1038/s41598-019-43922-z

**Published:** 2019-05-16

**Authors:** John A. Reid, Xavier-Lewis Palmer, Peter A. Mollica, Nicole Northam, Patrick C. Sachs, Robert D. Bruno

**Affiliations:** 1 Biomedical Engineering Institute, Old Dominion University, Norfolk, Virginia 23529 USA; 2 School of Medical Diagnostic & Translational Sciences, Old Dominion University, Norfolk, Virginia 23529 USA; 3 Molecular Diagnostics Laboratory, Sentar Norfolk General Hopsital, Norfolk, VA 23507 USA; 4 Biomedical Sciences Graduate Program, Eastern Virginia Medical School, Norfolk, Virginia 23501 USA

**Keywords:** Biotechnology, Cancer, Cell biology

## Abstract

The normal mammary microenvironment can suppress tumorigenesis and redirect cancer cells to adopt a normal mammary epithelial cell fate *in vivo*. Understanding of this phenomenon offers great promise for novel treatment and detection strategies in cancer, but current model systems make mechanistic insights into the process difficult. We have recently described a low-cost bioprinting platform designed to be accessible for basic cell biology laboratories. Here we report the use of this system for the study of tumorigenesis and microenvironmental redirection of breast cancer cells. We show our bioprinter significantly increases tumoroid formation in 3D collagen gels and allows for precise generation of tumoroid arrays. We also demonstrate that we can mimic published *in vivo* findings by co-printing cancer cells along with normal mammary epithelial cells to generate chimeric organoids. These chimeric organoids contain cancer cells that take part in normal luminal formation. Furthermore, we show for the first time that cancer cells within chimeric structures have a significant increase in 5-hydroxymethylcytosine levels as compared to bioprinted tumoroids. These results demonstrate the capacity of our 3D bioprinting platform to study tumorigenesis and microenvironmental control of breast cancer and highlight a novel mechanistic insight into the process of microenvironmental control of cancer.

## Introduction

Understanding the capacity of the local microenvironment (niche) to control the fate of cells is of vital importance to developmental biology, cancer biology, and regenerative medicine^1–4^. To this end, our group and colleagues have previously demonstrated that the regenerating mouse mammary gland can direct stem cells of non-mammary origin to a mammary epithelial cell fate *in vivo*^1,5–15^. The capacity of the local microenvironment to control differentiation extends to cancer cells as well^6,10,15,16^. Specifically, the regenerating mouse mammary gland directs normal mammary epithelial differentiation of human NTERA-2 teratocarcinoma^6^, human breast cancer^15^, and mouse MMTV-Erb2 mammary cancer cells^10^. For these studies, normal mouse mammary epithelial cells (MECs) were mixed with non-mammary or cancer cells and co-injected into the epithelial divested mammary fat-pads of recipient mice. The resulting glands contained chimeric epithelial trees consisting of both normal MECs and redirected non-mammary/cancer-derived cells. The redirected cells displayed normal MEC morphology and function and could self-renew and contribute to second generation outgrowths demonstrating they had not been terminally differentiated. These results were interpreted to mean that the non-mammary/cancer cells would be incorporated into niches as they were reformed by the dispersed MECs during transplantation. Once incorporated, they would adopt the function of that niche and therefore contribute to the regenerating gland by producing functional mammary epithelial progeny.

Mechanistic understanding of the capacity of the local microenvironment to direct the fate of cancer cells is important for cancer therapy and diagnostics. However, limitations of the *in vivo* model render such insights difficult to achieve. These limitations include low efficiency, low throughput, lack of an all human system, and limitations in experimental manipulation and cellular control. A complementary *in vitro* model system that allowed for precise control and reproducibility would therefore be beneficial. However, standard *in vitro* cell culture systems do not have the 3D architectures necessary to elicit the functional organization and cellular relationships of the *in vivo* environment^17^. For these reasons, 3D *in vitro* and *ex vivo* cell culture systems represent an indispensable tool to investigate the processes related to tissue and tumor formation. Unfortunately, current *in vitro* 3D models have many shortcomings, limiting their ability to investigate these processes^18^. For example, the overwhelming majority of these standard 3D systems rely on handheld-pipetting of premixed-ratios of cells with ECM substrates prior to gelling, or by manually blotting cell mixtures on top of a pre-formed ECM gel^19,20^. As a result, the distribution, size, morphology, and cell types within the resulting organoids vary greatly, which leads to difficulty in interpreting and reproducing experimental results^21^.

We have recently described the adaptation of a low-cost accessible 3D bioprinter for the purpose of precise cell printing within 3D hydrogels^22,23^. This bioprinting platform was designed for use in basic cell biology laboratories and can be used to generate large 3D mammary organoids in hydrogels^23^. Unlike traditional culture, the 3D bioprinted system precisely places cells allowing for greater control of organoid formation and experimental consistency. Here we describe the adaptation of our mammary epithelial organoid printing protocol for the generation of 3D tumoroids and chimeric organoids. We demonstrate that both MCF-7 and MDA-MB-468 human breast cancer cells incorporate into bioprinted organoids. We show that MCF-7 cells incorporated and contributed to luminal structure formation and undergo epigenetic alterations evidenced by significant increases in 5-hydroxymethylcytosine (5-hmC) levels. This system offers a significant improvement over traditional culture techniques and establishes a platform for future study into the microenvironmental control of cancer.

## Results

### Generation of patterned three-dimensional growth of mammary tumor cells

**Figure 1:**
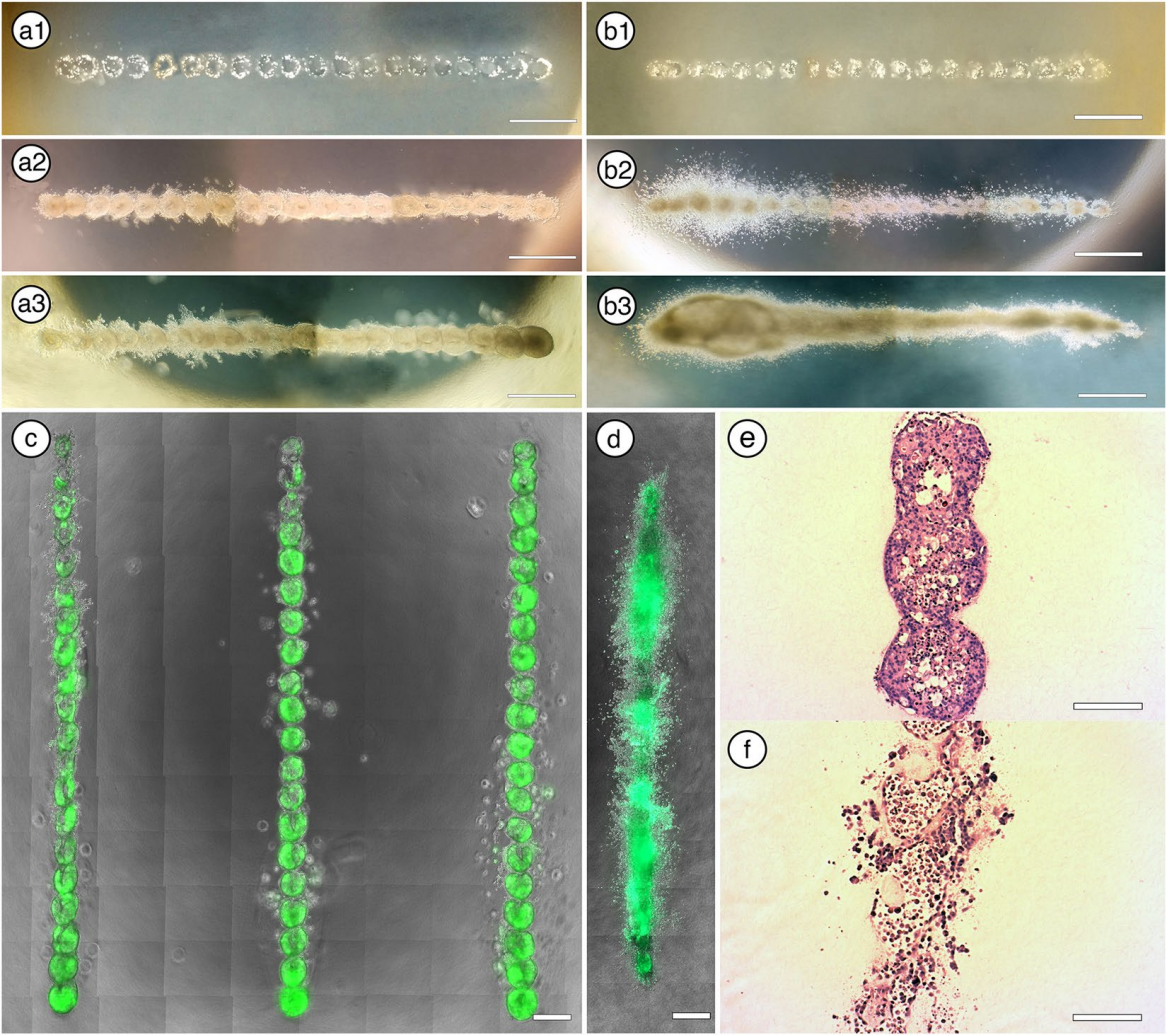
3D Bioprinting of consistent MCF-7 and MDA-MB-468 tumoroids. (**a1**–**3**) MCF-7 cell deposits (40 cells/deposit) spaced 300 µm apart at 1, 14, and 21 days post-printing. (**b1**–**b3**) MDA-MB-468 cell deposits (40 cells/deposit) spaced 300 µm apart at 1, 14, and 21 days post-printing. (**c**) Example of reliable printed array of GFP + MCF-7 tumoroids 21 days post-print with distinct structures. (**d**) Example of a linear array MDA-MB-468 tumor organoids at 21 days with all the multiple print sites fused into a single structure. (**e**,**f**) H&E stains of (**e**) MCF-7 and (**f**) MDA-MB-468 tumor organoids at 21 days. (Scale bars: a and b = 1 mm; c and d = 500 µm; e and f = 150 µm).

To determine the capacity of our bioprinting protocol to generate patterned 3D tumorigenic growths, we compared tumoroid formation efficiency in rat tail collagen gels between bioprinted and traditionally cultured green fluorescent protein (GFP) expressing MCF-7 and copGFP expressing MDA-MB-468 cells. MCF-7 and MDA-MB-468 represent luminal A and basal sub-types of breast cancer^24^. Our bioprinting method uses CNC processes to controllably-deposit cells in 3D locations of polymerized collagen I gels^22^. We bioprinted clusters of 40 tumor cells into equally spaced locations 300 µm apart (Fig. 1). The standard culturing protocol involves embedding dispersed cells into the hydrogel prior to polymerization. This method proved to be inefficient at generating tumoroid structures in collagen hydrogels. We quantitated the process by determining the frequency of wells that contained tumoroids (defined as cell clusters with volumes >0.001 mm^3^) between printed and traditionally cultured protocols. With traditional methods, MCF-7s and MDA-MB-468 cells never formed tumoroid structures (0/10 wells each, 2400 cells/well). Conversely, bioprinting was significantly more efficient (p < 0.0001 by Fisher’s Exact Test), resulting in 100% efficiency (10/10 wells), with a printing efficiency of 95% (57/60 prints, 40 cells/print, 60 prints/well).

**Figure 2:**
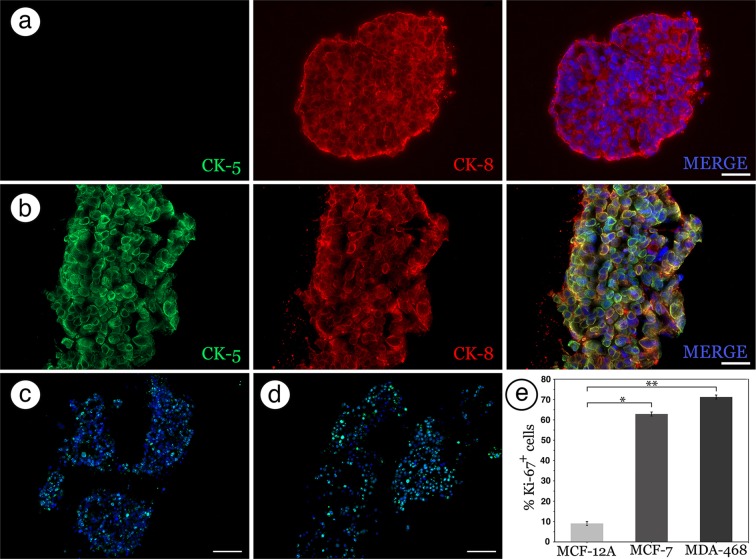
Bioprinted tumoroids maintain expected cytokeratin expression and high percentage of proliferative cells. (**a**,**b**) MCF-7 tumoroids stained negative for CK-5 (green) and positive for CK-8 (red). B) MDA-MB-468 tumoroids stained positive for both CK-5 (green) and CK-8 (red). (**c**,**d**) Ki67 staining of MCF-7 (**c**) and MDA-MB-468 (**d**) tumoroids. (**e**) Quantitation of ki67+ MCF-7 and MDA-MB-468 cells compared to MCF-12A cells. *p < 0.05; **p < 0.01. (Scale bars: a and b = 50 µm, c and = 100 µm).

Our bioprinting assay identified a discrepancy between the growth morphologies of the two tumor cell lines throughout the 21-day culture period. MCF-7 tumor cells formed compact, sphere-like structures with little evidence of coordinated growth among neighboring organoids, indicative of their previously known, non-invasive character (Fig. 1a,c,e)^25^. This allowed for patterned arrays of MCF-7 tumoroids to be printed (Fig. 1c). On the other hand, MDA-MB-468 cell growth illustrated the opposite effect, where invasive tumor cells equally dispersed into all radial directions of the rat tail collagen matrix, which resulted in a large, disordered structure lacking defined boundaries (Fig. 1b,d,f). These results are consistent with previous findings of enhanced invasive behavior of MDA-MB-468 cells^25^. Importantly, efficiency of tumoroid formation and organization was not impacted by modifications to print distances or cell number, so no additional optimization was undertaken. Our bioprinting device was designed to place minimal shear force on the cells and thus minimally impact their biology^22^. To confirm that the bioprinting process was not altering the phenotype of the cancer cells, we immunostained for cytokeratins 5 and 8 (CK5 and CK8). After bioprinting and tumoroid formation, MCF-7 cells remained CK8 positive and CK5 negative (Fig. 2a) consistent with their luminal phenotype^24^; MDA-MB-468 cells remained dual positive for CK5 and CK8 consistent with previous reports^24–26^. Furthermore, after 21 days in culture, the two tumor lines had high percentages of ki67+ cells, a marker of proliferation (Fig. 2c,d). The percentage of ki67+ MCF-7 and MDA-MB-64 tumor cells was significantly greater than bioprinted non-tumorigenic MCF12a cells (P < 0.01; Fig. 2e). These results demonstrate the capacity of the bioprinter to efficiently and consistently generate tumoroid structures in hydrogels without disrupting the phenotype of the printed cells. This simple, accessible, and inexpensive system thus offers a superior methodology for standardization of tumoroid related assays over traditional culture methodologies.

### Generation of chimeric structures in a 3D Gel

**Figure 3:**
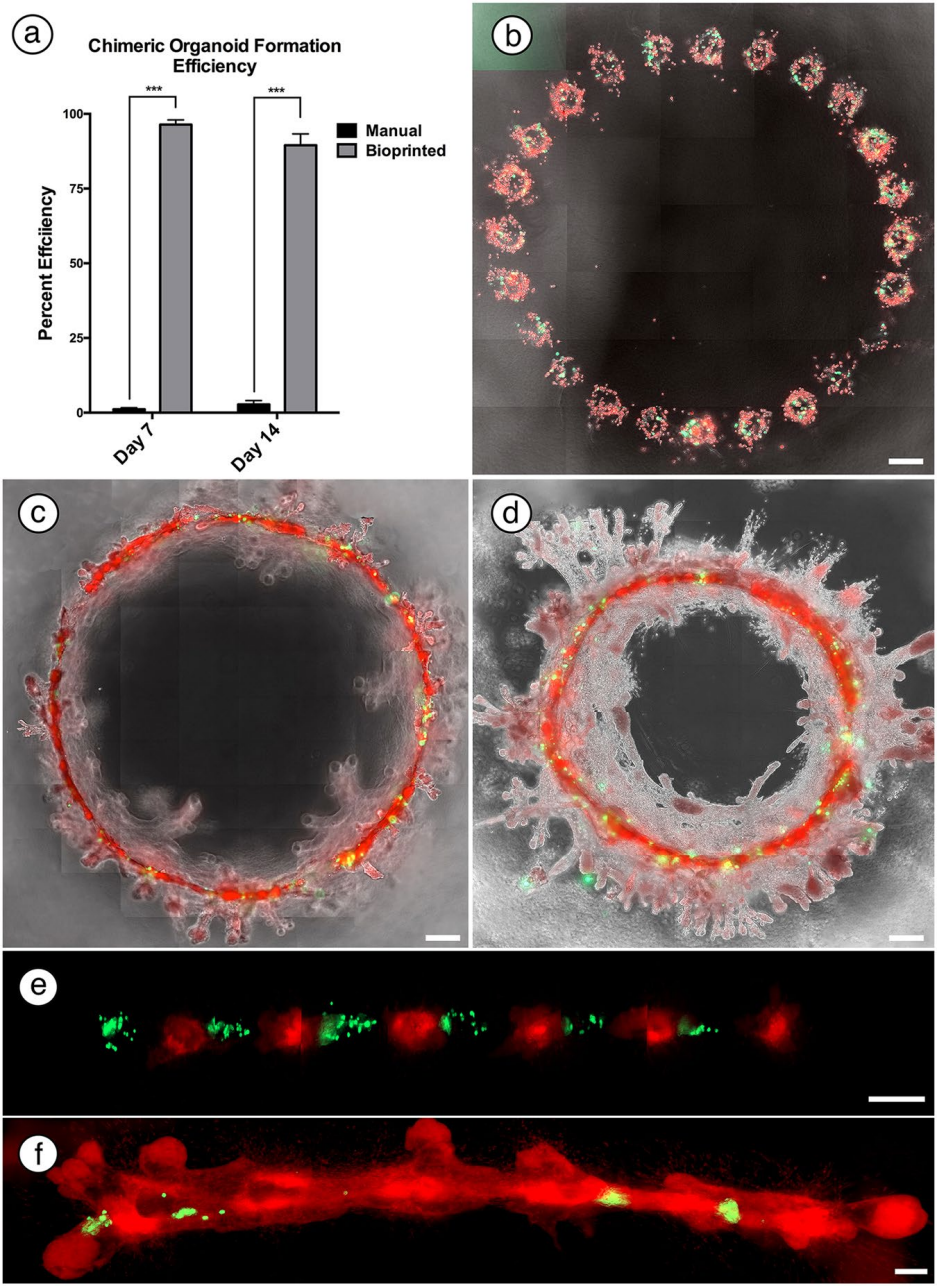
Generation of chimeric organoids using a 3D bioprinter. (**a**) Chimeric organoid formation was significantly improved by use of the 3D bioprinter compared to standard culture methods. ***p < 0.001. (**b**–**d**) Example of a typical large chimeric organoid generated by a 500 µm spaced print of a circle print pattern of a 5:1 ratio of MCF-12A (red) and MDA-MB-468 (green) cells at day 3 (**b**), day 7 (**c**) and day 21 (**d**). (**e**,**f**) Example of 300 µm spaced alternating prints of tumorigenic MDA-MB-468 cells (green) and MCF-12A cells (red) at day 1 (**e**) and day 7 (**f**) demonstrating incorporation of cancer cells into the organoid. (Scale bars: b–d = 500 µm; e and f = 200 µm).

Given the need to develop 3D *in vitro* models to investigate the molecular mechanisms underlying microenvironmental control of cancer cells, we began by determining the effectiveness of manual cell-matrix embedding to generate chimeric cell-organoids. Consistent with previous *in vivo* demonstrations, our *in vitro* chimera studies used a 5:1 ratio of normal to tumorigenic cells^1^. Thus, we mixed 5000 red fluorescent protein (RFP) labeled non-tumorigenic MCF-12A cells with 1000 cells from a single GFP tumorigenic cell line in neutralized rat-tail collagen and pipetted into wells of a 24 well plate and allowed the gel to polymerize. Under these conditions, MECs and tumorigenic cell lines were rarely able to generate chimeric organoid structures during the 21-day culture period (Fig. 3a). Analysis of the limited quantities of chimeric structures were further complicated by the excessive number and random distribution of MCF-12A organoids and persistence of unincorporated cancer cells within the hydrogel. This precluded any in-depth analysis of the redirected cells, as incorporation events were too sparse to facilitate any cellular or molecular analysis of redirection. However, random events did lead to occasional incorporation of cancer cells into chimeric structures (Supp. Fig. 1), which demonstrated the feasibility of a 3D *in vitro* model system.

To improve efficiency of chimeric structure formation, we next sought to guide the formation of chimeric organoids using our custom bioprinting system. We previously described our ability to standardize the frequency of organoid formation through control of the initial cell quantities within bioprinted cell-deposits; cell-deposits containing at least 40 cells formed organoids within 7 days post-printing^23^. Therefore, we mixed MCF12A cells and tumorigenic MCF-7 or MDA-MB-468 cells in a 5:1 ratio as above, and then printed 66 nl of cell mixtures equivalent to 40 cells in equally-spaced linear (300 µm) and circular (500 µm) arrays inside collagen I gels. Unlike the random cell distribution of manual embedding, our bioprinting method maintained GFP-labeled tumorigenic cells within the immediate vicinity of RFP-labeled MCF12A cells post-printing (Fig. 3b). Chimeric structures formed rapidly, with large contiguous chimeric organoids obvious by day 7 (Fig. 3c). After 3 weeks in culture, the cell clusters formed into large, contiguous epithelial structures containing chimeric constituents from both cancer cell lines (Fig. 3d). Interestingly, time-lapse imaging indicated both MCF-7 and MDA-MB-468 tumorigenic cell lines interact with MCF-12A cells, and actively migrate inside MCF-12A organoids (Sup. Movies 1 and 2). This is consistent with the activity of non-tumorigenic MCF-12A cells in previous findings which move between print locations and continue to display movement within organoids^23^. Importantly, as these structures began to generate branched extensions, tumor cells remained equally dispersed within the networked structures (Fig. 3), and very rarely were cancer cells found in the hydrogel unincorporated into the chimeric organoid. We could print these chimeric structures in either linear or circular arrays, consistent with previous findings that showed both array configurations could yield large contiguous organoids^23^.

We next quantitatively compared manual embedding vs our 3D bioprinted method to determine the efficiency of chimeric organoid formation. Surveys of manually-embedded gels indicated the initial 5000 MCF-12A cell-quantity resulted in a total of 929 ± 265 and 1060 ± 209 MCF-12A organoids at 7 and 14 days. Given the 5:1 ratio of tumorigenic cells in the initial cell mixtures of chimeric experiments, we expected to observe tumorigenic cells among 200 of the 1000 MCF-12A organoids in the embedded gels. Yet, among these organoids, only 2.3 ± 0.5 and 5.5 ± 1.3 chimeric organoids were observed at 7 and 14 days, respectively. Thus, generating chimeric organoids using manual embedding equaled a success rate of 1.15% and 2.75% at 7 and 14 days, respectively (Fig. 3a). Among experiments with 36 bioprinted cell-deposits, 34.7 ± 1.6 and 32.2 ± 3.8 chimeric cell-organoids formed at 7 and 14 days, respectively. Thus, bioprinted cell mixtures corresponded to a 96.4% and 89.5% chimeric organoid formation frequency at 7 and 14 days (Fig. 3a). When compared to manual methods, the number of bioprinted chimeric organoids increased significantly after both 1 week (p < 0.001) and 2 weeks (p < 0.01). Overall, this data highlights the increased efficiency of our 3D bioprinter to generate chimeric organoid-structures compared to manual matrix embedding procedures.

In addition to increased efficiency, our bioprinting device also provides the ability to generate unique combinations, geometric configurations, and temporal additions of multiple cell types. Previously, we described organoid fusion events where neighboring MECs initiate the formation of directional extensions to generate organized large epithelial structures^23^. Presumably, the areas where these restructuring processes occur contain ‘normal’ developmental cues. To determine if human cancer cell organoids could be influenced by these interactions, we used our bioprinting apparatus to place tumor-only cell-deposits between MCF12A cell-deposits in equally spaced (300 µM), linear arrays (Fig. 3e). MDA-MB-468 cells located in-between normal organoids were incorporated into large, chimeric organoid structures and failed to produce any tumoroid structures on their own (Fig. 3f). This demonstrates that mixed ‘ink’ is not required for chimeric structure formation, which adds to the versatility of the system.

**Figure 4:**
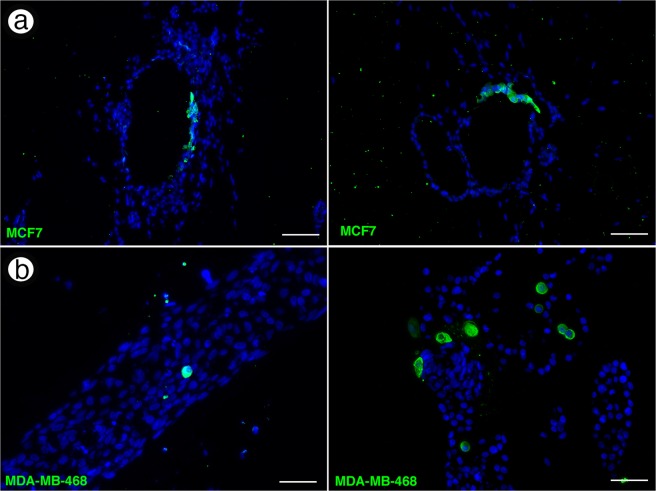
Incorporation of MCF-7 and MDA-MB-468 cells into chimeric organoids. (**a**,**b**) Immunofluorescence staining of 5 µm cross-sections of bioprinted chimeras with anti-GFP antibodies (green) reveals incorporation of MCF-7 (**a**) and MDA-MB-468 (**b**) cells. MCF-7 cells appeared in clusters contributing to luminal formation while MDA-MB-468 cells remained as single cells and were not observed lining the luminal layer. Sections are counterstained with DAPI (blue). All samples were analyzed 21 days post-print. Scale bars = 50 µm.

Staining of 5 µm cross-sections of bioprinted chimeric organoids for GFP confirmed incorporation of the tumorigenic cells into the MCF12a organoids (Fig. 4a,b). Notably, GFP+ MCF-7 cells were found to take part in luminal formation, appearing in linear groups of cells alongside GFP- MCF12A cells lining lumens (Fig. 4a), which was never observed when MCF-7 cells were bioprinted alone (Fig. 1). GFP+ MDA-MB-468s were also found integrated within a single 5 µm plane of MCF12A organoids (Fig. 4b). In general, MDA-MB-468 cells appeared as dispersed single cells rather than clusters, and we did not observe them lining lumens. Together, these results indicate our bioprinting process effectively generates chimeric structures, which holds the potential to mimic the incorporation of cancer cells within normal mammary structures previously described *in vivo*^1,2,6–11,13–15^.

### Integration into chimeric organoids causes increase in 5-hmC levels in MCF-7 Cells

**Figure 5:**
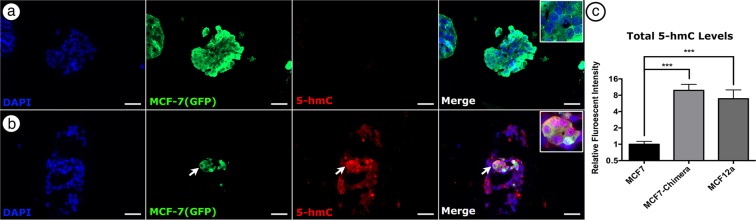
Incorporation into chimeric organoids significantly increases 5-hmC levels in MCF-7 cells. (**a**) Representative image of bioprinted MCF-7 cells (green) containing low levels of 5-hmC 21-days post-print. (**b**) Representative image of a chimeric organoid 21-days post-print containing MCF—7 (green) cells containing increased levels of 5-hmC (red) than when printed alone. Inset images in merged panel show higher magnification of the region of interest demonstrating absence or presence of 5-hmC in the nucleus. (**c**) Graph of the mean fluorescent intensity of 3 independent samples measured across 9 sections demonstrating incorporation of MCF-7 cells into chimeric organoids significantly increases (***p < 0.001) their 5-hmC levels, normalizing them to that seen in MCF12A cells. Scale bars = 50 µm.

To validate our model system as a methodology for studying molecular basis of cancer cell redirection, we next sought to determine if cancer cells integrated into 3D organanoids underwent epigenetic alterations mediated by their microenvironment. As the cancer cells retain genetic abnormalities in the chimeric structures *in vivo*^6^, changes in function are likely epigenetically mediated. It has been shown that cancer cells have reduced levels of 5-hmC^27^. 5-hmC is an intermediary in active demethylation process and can be detected *in situ*. We therefore explored whether incorporation in chimeric structures *in vitro* would alter 5-hmC levels in cancer cells. 21 days post-print, we confirmed bioprinted MCF-7 cells had significantly less 5-hmC than non-tumorigenic MCF-12A by immunofluorescence (Fig. 5). When incorporated into chimeric organoids however, MCF-7 cells had a significant increase in 5-hmC levels, up to that seen in non-tumorigenic MCF12A cells (Fig. 5b,c). This supports a model where incorporation into a normal mammary microenvironment mediates epigenetic alterations in the cancer cells, allowing for them to take part in normal morphological structures. In this way, this work perfectly mimics our results *in vivo*, and underscores the utility of our system for studying microenvironmental control of cancer cells.

## Discussion

This work provides a description of how our bioprinting technology can be used to standardize current and future chimeric models of 3D epithelial cell culture assays. We demonstrate the superiority of our bioprinting system over conventional manual embedding methods for studying tumor cell behaviors, particularly in the context of cancer cell redirection. Our low cost, open source, and accessible system is superior to traditional culture methods in generating controllable, precise tumoroid arrays for assay standardization. While many bioprinting apparatuses have been described that offer more robust and complex printing dynamics^28^, these systems are often beyond the reach of traditional cancer laboratories. Our simplistic design of an open source system that can be downloaded (www.odustemcell.org), printed and used in any laboratory improves upon the widely used traditional 3D culture assays to increase efficiency and reproducibility and allow for standardization of assays across laboratories. In addition, this system can be adapted for various drug screening and clonogenic stem cell associated assays.

Our laboratory has interest in the role of the microenvironment in controlling cancer cell behavior. The model system described here is the first system to allow robust analysis of this process *in vitro*. We believe the major advantage of our system in this context is the ability to print localized concentrations of mixed cell populations at equal distances. In random culture techniques, chimeric formation is likely limited by cellular concentration. Too many cells limits the formation of 3D structures, while too few cells limits the probability of chimeric structures forming. However, by condensing cells within a small 3D location (<100 nl of extruded fluid), we can get efficient chimeric structure formation. In these chimeric structures the cancer derived cells took part in normal organoid formation, consistent with previous *in vivo* results. While we did not specifically quantitate the process, we noted that the ratio of cancer to normal cells decreased in the fully formed structures. This is consistent with the interpretation that the normal microenvironment suppresses the growth of the cancer cells. This platform also allows for future studies into the temporal and spatial constrictions of cancer cell redirection. As demonstrated in Fig. 3e,f, cancer cells can be printed near normal cells in separate injections. This will allow for analysis of the effect of placing tumor cells around or near pre-formed organoids to help decipher differential effects of signaling during organoid development versus maintenance. The efficiency of cancer cell integration in the chimeric studies also allows for ease in isolation, without concern for contamination from unincorporated cancer cells. This will allow for fluorescent activated cell sorting of chimeras. Furthermore, because of the capacity of the printer to efficiently generate chimeric structures, future studies will explore the transplantability of 3D bioprinted chimeric organoids into cleared mammary fat pads. This would greatly increase the efficiency of the *in vivo* model.

In addition, this report is the first to demonstrate a change in 5-hmC levels in cancer cells upon redirection by a normal microenvironment. This finding is particularly interesting given recent results that linked EGFR mediated MAPK signaling to decreases in 5-hmC levels in cancer cells^29^. It was also previously shown that Erb2 phosphorylation in MMTV-Erb2 mouse mammary tumor cells was suppressed during chimeric gland formation *in vivo* or during co-culture *in vitro*^10^. Future studies using the 3D bioprinted approach outlined here can investigate this potentially important mechanistic insight.

We recently describe the capacity of normal murine mammary ECM to direct differentiation of testicular and embryonic stem cells inside the mouse mammary gland^30^. The 3D bioprinting technology outlined here can also facilitate well controlled future studies aimed at exploring the role of the normal ECM in directing cell fate. This has the potential to develop not only mechanistic insights into the role of the ECM in controlling cell fate, but also for the development of all-human biomimetic culture systems. Future studies will also take advantage of the system to manipulate additional parameters such as localized gel stiffness to explore the role of mechanical contributions to cancer cell redirection.

Understanding the bidirectional communication between tumor cells and their microenvironment represents a powerful, advantageous way to investigate the mechanisms that influence disease promotion and progression. Furthermore, identifying the contextual contributions related to normalizing or reversing the tumor-specific ECM associated with cancer stands as an interesting target for novel screening methods and therapeutic targets for clinical tumor therapy. The system described here offers a novel, high-throughput, and reproducible experimental strategy to study this phenomenon. Furthermore, this study provides a foundation for the observations made in the mammary gland to be tested in other cellular systems. This is facilitated by the open source nature of our printing protocols, which can easily be adapted to any 3D culture system. This offers our laboratory and others interested in this field the ability to develop experiments built off the printing protocols outlined here.

The capacity of normal cells to produce microenvironments (niches) capable of controlling the fate of cancer cells offers a unique insight into the biology of cancer. To study this, more robust model systems are required. The process described here, the adaptation of a low-cost and accessible bioprinting system for the generation of normal/tumor chimeras, achieves this goal. Our system efficiently generates normal human mammary organoids with cancer cells incorporated throughout, mimicking published *in vivo* findings in mice. This process was used to identify changes in 5-hmC levels within cancer cells incorporated into chimeras, consistent with a model where the normal microenvironment mediates epigenetic changes to redirect the cancer cells away from a tumorigenic fate. Thus, our bioprinting platform allows for mechanistic insight into the process of cancer cell redirection, and serves as an important platform for future studies into the process.

## Methods

### Cell culture

The immortalized non-tumorigenic human breast epithelial cell line, MCF-12A, and the breast carcinoma cell lines MCF-7 and MDA-MB-468 were purchased from American Type Culture Collection (ATCC). MCF-12A cells are considered a model for normal human MECs. The MCF-7 cell line represents a rapidly growing, luminal carcinoma cell line that is estrogen receptor-positive and non-invasive^24,31^. MDA-MB-468 cells are a basal, EGF receptor rich, estrogen receptor-negative and invasive breast cancer cell line^24,25,32^. All cells were stably transduced with premade lentiviral vectors to express the following: MCF-12A cells expressed RFP form the CMV enhancer/chicken β-actin promoter (CAG; Cellomics Technology, MD, USA); MCF-7 cells expressed GFP from the eIF promoter (Cellomics Technology) and MDA-MB-468 cells expressed copGFP (TurboGFP) from the eIF promoter (Systems Biosciences).

All cells were maintained on 2D tissue culture plastic prior to printing. MCF-12A cells grown in DMEM/F12, 5% Horse Serum, 20 ng ml^−1^ hEGF, 0.01 mg ml^−1^ bovine insulin, 500 ng ml^−1^ hydrocortisone and 1% ABAM (all purchased from ThermoFisher). MCF-7 and MDA-MB-468 cells were maintained in Dulbecco’s Modified Eagle’s Medium supplemented with 10% fetal bovine serum (FBS, Life Technologies) and 1% Antibiotic/Antimycotic(ThermoFisher). All cells were cultured at 37.0 °C and 5.0% CO_2_. After 80% confluence, the cells were dissociated using TrypleE (ThermoFisher) and collected by centrifugation. Chimeric organoids were cultured using DMEM/F12, 5% Horse Serum, 20 ng ml^−1^ hEGF, 0.01 mg ml^−1^ bovine insulin, 500 ng ml^−1^ hydrocortisone and 1% Antibiotic/Antimycotic (ThermoFisher).

### Preparation of 3D collagen ECMs

For both cell-matrix manual embedding and 3D bioprinting experiments, 3D rat-tail collagen gels were made according to manufacture protocols. Briefly, with all materials on ice, rat-tail collagen (Corning) was diluted to a final concentration of 1.3 mg/ml with the addition of 1X PBS, and 1 N NaOH to a neutral pH. To polymerize gels, the 4 °C neutralized rat-tail collagen was then dispensed into multi-well plates and then incubated at 37 C for 60 minutes.

### Manual Cell-matrix embedding

For epithelial cell only manual cell-matrix embedding studies, single cell suspensions of cells were mixed with 4 °C 1.3 mg/ml final concentration neutralized rat tail collagen I (Corning) as described previously^23^. Mixtures were then pipetted into a 24 well plate and allowed to solidify for 1 hour in a laboratory incubator at 37.0 °C. After gelation (solidification), 500 µl of cell appropriate cell media was added to the wells. For chimeric studies, MCF-12A cells were mixed with one of the two tumorigenic cell lines at a 5:1 ratio. Immediately after mixing with neutralized rat-tail collagen, 500 µl gel material containing 2400 (cancer cells alone) or 6000 (chimeric studies) cells was dispensed into a 24 well plate and allowed to solidify as described above.

### 3D bioprinting

The 3D bioprinter used in these studies is a fully customized Felix 3.0 described previously^22^. Our 3D bioprinter is used to robotically dispense various cells into specified 3D locations of a polymerized collagen gel^22,23^. The 3D files to manufacture our bioprinter are freely available from our website (www.odustemcell.org). It uses a microstepper motor joined to a fine-resolution leadscrew which controls a plunger inserted into the rear opening of a glass pulled micropipette (Drummond Science Company). All glass pulled pipettes were manufactured on a Sutter P97 programmable pipette puller and are standardized to a tip diameter of 50 μm. Printing operations were all performed as previously described^22,23^. Briefly, neutralized collagen I gel material was dispensed into a 24 well plate as described above. To manufacture the ‘bio-Ink’, MCF-12A, MCF-7 and MDA-MB-468 cells were dissociated into single cells using TrypleE (ThermoFisher), centrifuged at 300 × g, and re-suspended in media to a concentration of 60 × 10^4^ cells ml^−1^. For chimeric experiments, cells were then mixed to a 5:1 ratio and loaded into the injection device affixed to the printer. Printing operations were optimized to extrude specified numbers of cells (40 cells/injection event) inside the collagen I gel via a CNC insertion routine which deposited cell containing media (total of 66 nl/injection event) at a specified ‘target’ location inside the 24-well dish containing the already polymerized collagen I gel. Users specified parameters are set via a custom MATLAB program, which sets the number of intended wells, printing locations, distances among printing locations, and the number of cells per target location. The subsequently outputted G-code CNC routine is then loaded into a free-ware printer control software (Repetier Host), and the routine is then initiated in the printer following the loading of the ‘bio-ink’ into the injection device. The bioprinting system was located inside a benchtop biosafety cabinet during all printing operations. The heated print bed was set to 37 °C for all printing operations.

### Histology and immunofluorescence Staining

Unless otherwise noted, samples were fixed in 10% NBF 21-days post-printing. Paraffin embedded sections were processed using standard immunohistochemistry protocols. All sections underwent heat-mediated antigen retrieval using pH 9 Tris-EDTA with 0.05% tween 20 prior to staining. Primary antibody incubations were performed in a humidified chamber at 4 °C overnight (16 hrs). Secondary antibody incubations were performed at room temperature for 1 hr. All sections were counterstained with DAPI and imaged using a Zeiss Axio-observer Z1 fluorescent microscope. Image processing was completed with Zeiss Zen software.

The following primary antibodies were used: anti-green fluorescent protein rabbit IgG Alexafluor 488 conjugated (1:75; Invitrogen A21311); chicken anti-GFP (1:1000; Abcam ab13970), rabbit anti-Turbo GFP (1:1000; ThermoFisher PA5-22688); rabbit monoclonal antibody to cytokeratin 5 [EP1601Y] (1:75; ab52635, Abcam), mouse monoclonal antibody to cytokeratin 8 [C-51] (1:35; ab2531, Abcam), rabbit monoclonal anti-5-hmC [RM236] (1:50; ThermoFisher MA5-24695). Appropriate Alexafluor 488 or 568 conjugated secondary antibodies (1:1000; ThermoFisher) were used for visualization.

For 5-hmC quantitation, fluorescent intensity was quantified within cell nuclei using Zeiss Zen software. 5-hmC levels were measured in MCF-7 cells grown alone as tumoroids, MCF12A cells growing as organoids, and MCF-7 cells growing within normal organoids with MCF12A cells (chimeras). In chimeras, MCF-7 cells were identified by co-staining with an anti-GFP antibody. Cells were measured from multiple sections across 3 independent experiments. Individual cell measurements were then averaged for statistical analysis.

### Statistical analysis

Values represent mean ± standard deviation of samples. Data represent three or more independent experiments. Tumoroid formation rates were compared using a Fisher’s exact test. Fluorescent intensity of 5-hmC and percentage of ki67+ cells were compared by a one-way ANOVA with a Dunnett’s post-hoc. Efficiencies of chimeric gland formation were compared by a two-way ANOVA.

## Supplementary information

Supplemental movie 1

Supplemental movie 2

Supplementary Data

## Data Availability

The datasets generated during and/or analyzed during the current study are available from the corresponding authors on reasonable request.
