# Supplementary material for: A 3D bioprinter platform for mechanistic analysis of tumoroids and chimeric mammary organoids
Source: Sci Rep. 2019 May 16;9:7466. doi: 10.1038/s41598-019-43922-z (PMC6522494; doi:10.1038/s41598-019-43922-z)
Supplement: Supplementary file 3 — Supplementary Data [file 41598_2019_43922_MOESM3_ESM.pdf]

Supplemental Data: A 3D bioprinter platform for mechanistic analysis of tumoroids and chimeric mammary organoids

John A. Reid[a], Xavier-Lewis Palmer[a], Peter A. Mollica[b,c], Nicole Northam[d], Patrick C. Sachs[b*^], Robert D. Bruno[b*^]

[a]Biomedical Engineering Institute, Old Dominion University, Norfolk, Virginia, 23529
[b]School of Medical Diagnostic & Translational Sciences, Old Dominion University, Norfolk, Virginia, 23529
[c]Molecular Diagnostics Laboratory, Sentar Norfolk General Hopsital, Norfolk VA 23507
[d] Biomedical Sciences Graduate Program, Eastern Virginia Medical School, Norfolk, Virginia 23501

[^]Corresponding authors: Patrick C. Sachs psachs@odu.edu; Robert D. Bruno rbruno@odu.edu
*these authors contributed equally to the work

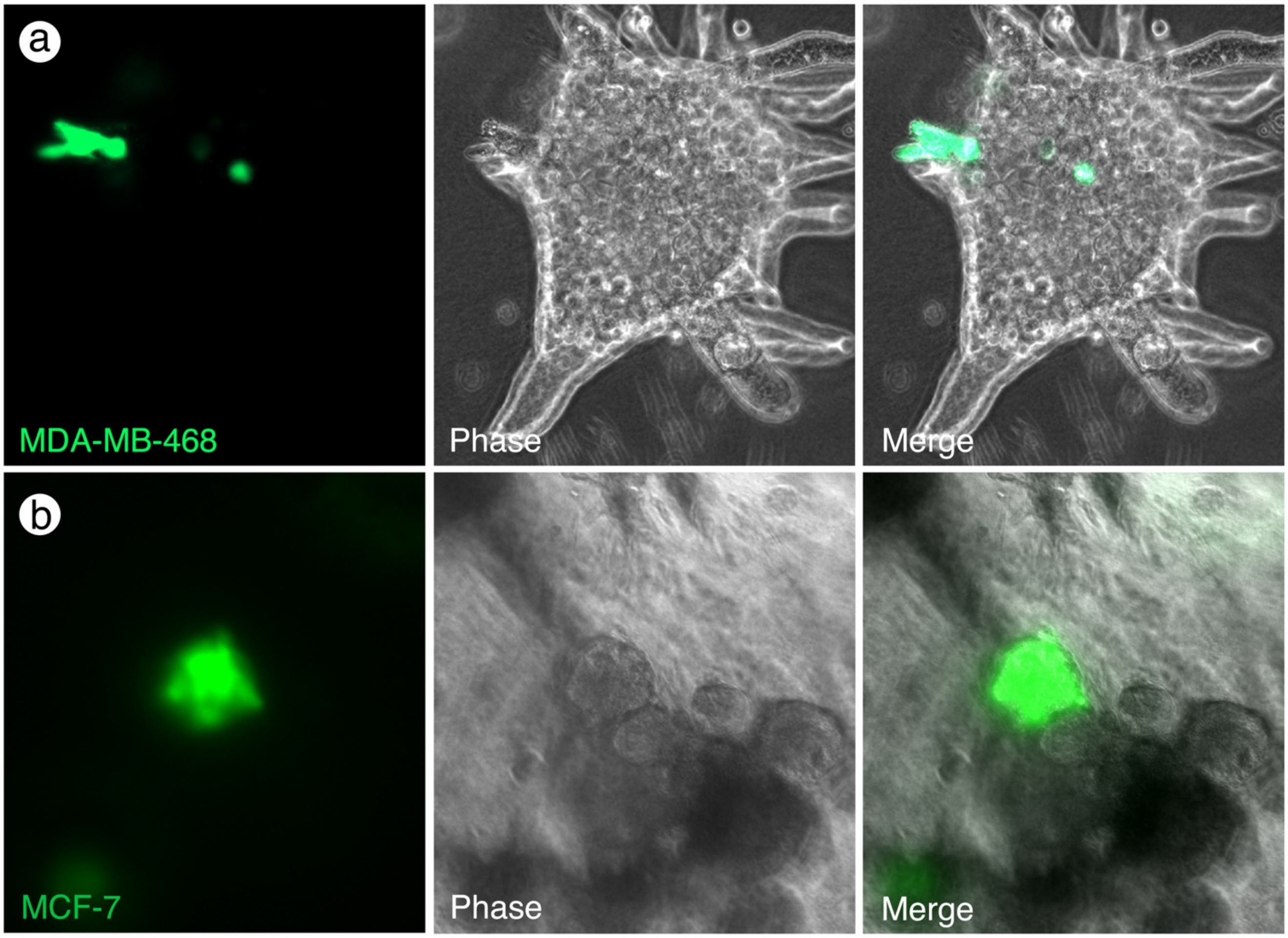

Supplemental Figure 1: *MDA-MB-468 and MCF-7 cells rarely integrate into normal Luminal like structures in random 3D hydrogels cultures.* GFP labeled MDA-MB-468 cells **(A)** and RFP labeled MCF-7 cells **(B)** were randomly mixed with non-RFP MCF12A cells in collagen hydrogels. Following 21 days of culture luminal structures with rare integration events (right panel) were imaged.

Supplemental Movie Legends:

Supplemental Movie 1: Time lapse imaging of MDA-MB-468 cells (green) within a MCF12A chimera

Supplemental Movie 2: Time lapse imaging of MCF-7 cells (green) within a MCF12A chimera
